# Fetal Growth Retardation and Lack of Hypotaurine in Ezrin Knockout Mice

**DOI:** 10.1371/journal.pone.0105423

**Published:** 2014-08-21

**Authors:** Tomohiro Nishimura, Kei Higuchi, Yoshimichi Sai, Yuki Sugita, Yuko Yoshida, Masatoshi Tomi, Masami Wada, Tomohiko Wakayama, Atsushi Tamura, Sachiko Tsukita, Tomoyoshi Soga, Emi Nakashima

**Affiliations:** 1 Division of Pharmaceutics, Faculty of Pharmacy, Keio University, Minato-ku, Tokyo, Japan; 2 Department of Pharmacy, Kanazawa University Hospital, Kanazawa, Japan; 3 Department of Histology and Embryology, Graduate School of Medical Sciences, Kanazawa University, Kanazawa, Japan; 4 Laboratory of Biological Science, Graduate School of Frontier Biosciences and Graduate School of Medicine, Osaka University, Suita, Osaka, Japan; 5 Institute for Advanced Biosciences, Keio University, Tsuruoka, Yamagata, Japan; Medical Faculty, Otto-von-Guericke University Magdeburg, Germany

## Abstract

Ezrin is a membrane-associated cytoplasmic protein that serves to link cell-membrane proteins with the actin-based cytoskeleton, and also plays a role in regulation of the functional activities of some transmembrane proteins. It is expressed in placental trophoblasts. We hypothesized that placental ezrin is involved in the supply of nutrients from mother to fetus, thereby influencing fetal growth. The aim of this study was firstly to clarify the effect of ezrin on fetal growth and secondly to determine whether knockout of ezrin is associated with decreased concentrations of serum and placental nutrients. Ezrin knockout mice (Ez^−/−^) were confirmed to exhibit fetal growth retardation. Metabolome analysis of fetal serum and placental extract of ezrin knockout mice by means of capillary electrophoresis–time-of-flight mass spectrometry revealed a markedly decreased concentration of hypotaurine, a precursor of taurine. However, placental levels of cysteine and cysteine sulfinic acid (precursors of hypotaurine) and taurine were not affected. Lack of hypotaurine in Ez^−/−^ mice was confirmed by liquid chromatography with tandem mass spectrometry. Administration of hypotaurine to heterogenous dams significantly decreased the placenta-to-maternal plasma ratio of hypotaurine in wild-type fetuses but only slightly decreased it in ezrin knockout fetuses, indicating that the uptake of hypotaurine from mother to placenta is saturable and that disruption of ezrin impairs the uptake of hypotaurine by placental trophoblasts. These results indicate that ezrin is required for uptake of hypotaurine from maternal serum by placental trophoblasts, and plays an important role in fetal growth.

## Introduction

In placenta, syncytiotrophoblasts form a continuous epithelial barrier and functionally regulate exchange of nutrients and waste products between the maternal and fetal circulations, namely, across the placental barrier. The placental barrier in humans is composed of a single layer of syncytiotrophoblast, which comprises two opposing surfaces, an apical surface bathed by the maternal circulation and a basolateral surface, which is adjacent to the fetoplacental circulation. Plasma membrane transporters at the apical membrane regulate transport of nutrients and xenobiotics between maternal blood and placenta [1]–[3]. On the other hand, rodents have a double layer of syncytiotrophoblast, I and II, and in this case the apical surface of syncytiotrophoblast I faces the maternal circulation, while the basolateral surface of syncytiotrophoblast II faces the fetal circulation. Proper function of transporters at the syncytiotrophoblast is considered to be critical for fetal growth, because decreased activity of placental amino acid transporters is associated with intrauterine growth retardation [4], [5].

Ezrin/radixin/moesin (ERM) proteins are membrane-associated cytoplasmic proteins that provide a regulated linkage between transmembrane proteins, including plasma membrane transporters, and cortical actin filaments. These linkages are crucial for organization and maintenance of specialized membrane domains, including the apical plasma membrane domain of epithelial cells [6], [7]. In addition, phosphorylation of ezrin plays a role in regulation of the functional activities of transmembrane proteins [8]. Recent studies indicate that the functional expression of some apical membrane transporters such as cystic fibrosis transmembrane conductance regulator (CFTR) and Na^+^/H^+^ exchanger (NHE3) is regulated by their interaction with ezrin in the kidney and respiratory tract [9]–[11]. Ezrin is the most abundant of the ERM proteins and is specifically associated with the apical membrane of syncytiotrophoblast [12]–[14]. Thus, transporter/ezrin complexes are considered to be important for the proper transport of physiological nutrients and metabolites in syncytiotrophoblast.

Homozygous ezrin-knockout (*Ez^−/−^*) neonates were born in a sub-Mendelian ratio after mating of heterozygous ezrin-knockout (*Ez^+/−^*) mice, and *Ez^−/−^* pups died before weaning [15], [16]. Defects in epithelial organization and villus morphogenesis were observed in the gastrointestinal tract of *Ez^−/−^* pups, and might lead to neonatal death. However, these defects are not likely to directly affect fetal growth, since fetuses take up nutrients from the maternal blood via the placenta. These considerations led us to hypothesize that ezrin at the apical membrane of syncytiotrophoblast contributes to fetal growth by supporting or regulating the functional expression of nutrient transporter(s).

Therefore, the aims of this study were firstly to confirm that fetal growth of *Ez^−/−^* mice is impaired, and secondly to examine whether this impairment is associated with dysfunction of nutrient transport at the placental syncytiotrophoblast. For this purpose, we need to know whether the concentrations of any key nutrients are decreased in *Ez^−/−^* mice. We examined this issue by means of metabolome analysis using capillary electrophoresis–time-of-flight mass spectrometry (CE-TOF/MS), which can quantify hundreds of physiological metabolites, including amino acids and their metabolites [17].

## Materials and Methods

### Chemicals and antibodies

Rabbit polyclonal anti-ezrin (H-276) antibody was purchased from Santa Cruz Biotechnology (Santa Cruz, CA, USA). Other chemicals were of analytical grade, and were purchased from Sigma (St. Louis, MO, USA) or Wako Pure Chemicals (Osaka, Japan).

### Animals

**Figure 1:**
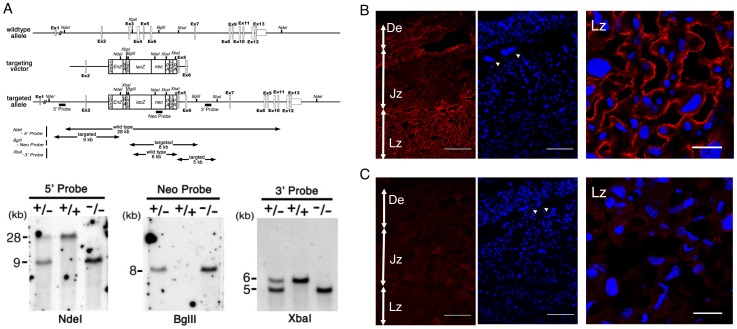
Placental distribution of ezrin and generation of *Ez* ^−/−^ mice. (A) Schematic representation of the wild-type allele, targeting vector, and targeted allele of the mouse ezrin gene (*Vil2*). The first ATG codon is located in exon 2. The targeting vector contained the neomycin-resistance cassette (neo) combined with engrailed-2 (En-2)/splicing acceptor (SA)/internal ribosome entry site (IRES)/β-galactosidase (lacZ) on its 5′ side and with a polyadenylation site (PA) on its 3′ side, which was designed to be inserted between exons 2 and 5 in the targeted allele. Southern blot analysis of genomic DNA from *Ez* ^+/+^, *Ez* ^+/−^, and *Ez* ^−/−^ mice. The NdeI fragments were detected by the 5′ probe from the wild-type (28 kb) and targeted alleles (9 kb), and the BglII fragment was detected by the neo probe from the targeted allele (8 kb). The XbaI fragments were detected by the 3′ probe from the wild-type (6 kb) and targeted alleles (5 kb). Immunofluorescence micrographs of frozen sections of *Ez* ^+/+^ (B) and *Ez* ^−/−^ (C) placenta at gestational day 18.5 obtained with rabbit polyclonal antibody against ezrin (red), showing complete loss of ezrin in *Ez* ^−/−^ placenta. Nuclei were stained with DAPI (blue). De, decidua; Jz, junctional zone; Lz, labyrinth zone. Arrowheads indicate trophoblast giant cells. Confocal microscopic images of ezrin in labyrinth area were also shown in right panels (B, C). Scale bars represent 100 µm (left, center) and 20 µm (right).

We isolated a mouse ezrin (*Vil2*) genomic clone of 13.7 kb that included exons 2–6 from a λ129/Sv mouse genomic library. The targeting vector (Figure 1B) was constructed by standard recombinant DNA techniques; the fragment containing exons 3–4 was deleted from the genomic clone and replaced by a neomycin-resistance cassette combined with the engrailed-2 splice receptor, the internal ribosome entry site, the β-galactosidase gene, a polyadenylation signal, loxP, and a polyadenylation signal. The vector was electroporated into J1 ES cells, which were plated onto feeder cells and selected with G418. G418-resistant colonies were collected and evaluated for homologous recombination by Southern blot analysis. Two clones were injected into C57BL/6 blastocysts, which were then transferred into the uteri of pseudopregnant ICR recipients. Male chimeras with extensive ES cell contributions to their coats were bred with C57BL/6 female mice. We genotyped tail DNA from agouti F1 offspring by Southern blotting. *Ez^+/−^* mice were interbred to produce *Ez^−/−^* mice. After overnight mating, we checked for vaginal plugs to confirm successful mating and defined the time as gestational day 0.5. All animal experiments were approved by the institutional animal care and use committees of the Faculty of Medicine, Kyoto University, and the Institutional Animal Care Committee, and complied with the standards set out in the Guideline for the Care and Use of Laboratory Animals in Keio University.

### Morphological and immunohistochemical analyses

At gestational day 18.5, pregnant mice were anesthetized with pentobarbital (Nembutal; Dainippon Pharmaceutical, Osaka, Japan). For morphological analysis, the placenta was isolated and soaked overnight in 4% paraformaldehyde in 0.1 M phosphate buffer at 4°C. The tissues were then dehydrated overnight in 20% sucrose/HBSS (Hanks balanced salt solution) and embedded in OCT (Optimal Cutting Temperature) compound. Tissue sections (4 µm) were prepared with a cryostat. The placental sections were stained with Mayer's Hematoxylin Solution (Wako Pure Chemicals) and Eosin Y (Wako Pure Chemicals) for general morphology. They were also stained with a terminal deoxynucleotidyl transferase dUTP nick end labeling (TUNEL) assay kit (In Situ Cell Death Detection Kit, TMR red, Roche Diagnostics, Basel, Switzerland) for analysis of apoptotic cells. For immunohistochemistry, the anesthetized mice were perfused transcardially with 4% paraformaldehyde in 0.1 M phosphate buffer. Placentas were dissected out and kept for 2 hr in the same buffer. Placenta sections were fixed with methanol, blocked, and then reacted with anti-ezrin antibody (H-276), followed by Alexa Fluor 594 goat anti-rabbit IgG (Life Technologies). The sections were sealed with Vectashield Hard set (Vector Laboratories, Burlingame, CA, USA) containing DAPI, and viewed with a fluorescence microscope (Nikon, Tokyo, Japan).

### Ultrastructure analysis by electron microscopy

*Ez^+/+^* and *Ez^−/−^* placentas were fixed in 2.5% glutaraldehyde and 2% formaldehyde in phosphate-buffered saline (PBS, pH 7.4). They were then fixed with 1% OsO_4_, stained with 1% uranyl acetate and embedded in Glicidether (Selva Feinbiochemica, Heidelberg, Germany). Ultrathin sections were prepared and subjected to observation with a Hitachi H-700 electron microscopy (Hitachi, Tokyo, Japan).

### Metabolome analysis

Quantitative analysis of charged metabolites in fetal plasma and placenta by capillary electrophoresis with time-of-flight mass spectrometry (CE-TOF-MS) was performed as reported [17]. Fetal plasma and placenta were collected from each fetus at gestation day 18.5 and pooled according to genotyping at −80°C. Wet weights of each fetus and placenta were measured after removal of fluid by blotting with absorbent paper. Metabolites in these samples were separated by CE on the basis of charge and size and selectively detected using MS by monitoring over a wide range of *m/z* value.

### Continuous administration of hypotaurine to pregnant mice

*Ez^+/−^* female mice were mated with *Ez^+/−^* male at night and the next day was defined as gestational day 0.5. Hypotaurine solubilized in phosphate-buffered saline (PBS) (500 mg/mL) was subcutaneously administered to the pregnant mice from gestational day 8.5 via an osmotic pump (ALZET, Cupertino, CA) with a flow rate of 1 µL/hr. Vehicle (PBS only) was administered to a negative control group. Maternal blood was intermittently sampled until gestational day 18.5, when each dam was sacrificed. Fetus and placenta were weighed, and placenta and fetal blood were sampled.

### Sample preparation for measurement of hypotaurine in plasma and tissues

Plasma was prepared by centrifugation of 15,000 rpm for 10 min at 4°C. A 5 µL aliquot of serum was mixed with 50 µL of 1500 ng/mL 2-aminoethyl hydrogen sulfate solution as an internal standard and 400 µL of 0.2 M citric acid buffer (pH 2.0) and the mixture was incubated at 4°C for 30 min. After centrifugation at 78,000 g for 20 min, 400 µL of the supernatant was neutralized by mixing with 100 µL of 8.4% ammonia solution. Weighed placenta was mixed with 10 volumes of ice-cold PBS and homogenized in an IKA T10 basic ULTRA-TURRAX (IKA-Works, Wilmington, NC) for 30 sec on ice. A 50 µL aliquot of the homogenate was mixed with 500 µL of 1500 ng/mL 2-aminoethyl hydrogen sulfate and centrifuged at 15,000 rpm for 10 min at 4°C. Then 110 µL of the supernatant was mixed with 800 µL of 0.2 M citric acid buffer (pH 2.0). The mixture was incubated at 4°C for 30 min, then centrifuged at 78,000 g for 20 min, and 400 µL of the supernatant was neutralized with 100 µL of 8.4% ammonium solution. All samples were filtered through a 0.45 µm PTFE membrane before quantitative determination of target molecules by LC/MS/MS.

### Measurement of hypotaurine by LC-MS/MS

The quantification of hypotaurine by liquid chromatography with tandem mass spectrometry (LC-MS/MS) was performed according to the reported method using 2-aminoethyl hydrogen sulfate as an internal standard [18]. The LC-MS/MS system was equipped with a constant flow pump (LC-20AD; Shimadzu, Kyoto, Japan), an automatic sample injector (SIL-20A HT; Shimadzu), a column oven (CTO-20A; Shimadzu), and a mass spectrometer (API 3200; Applied Biosystems, Foster City, CA, USA). The analytical column was HYPERCARB (Thermo Scientific, Waltham, MA, USA). The mobile phase was 10 mM ammonium acetate adjusted to pH 9.3, and the flow rate was 0.1 mL/min. Separation was performed at 50°C. The multiple reaction monitor was set at 108.0 to 64.0 m/z and 139.9 to 96.9 m/z for hypotaurine and 2-aminoethyl hydrogen sulfate, respectively, in the negative mode.

### Statistical analysis

The data were expressed as mean ± standard error of the mean (S.E.M.). The statistical significance of differences was determined by using Student's t-test or one-way ANOVA with Dunnett's post-hoc test. A P value of less than 0.05 was considered significant.

## Results

### Phenotype of *Ez^−/−^* mice

**Figure 2:**
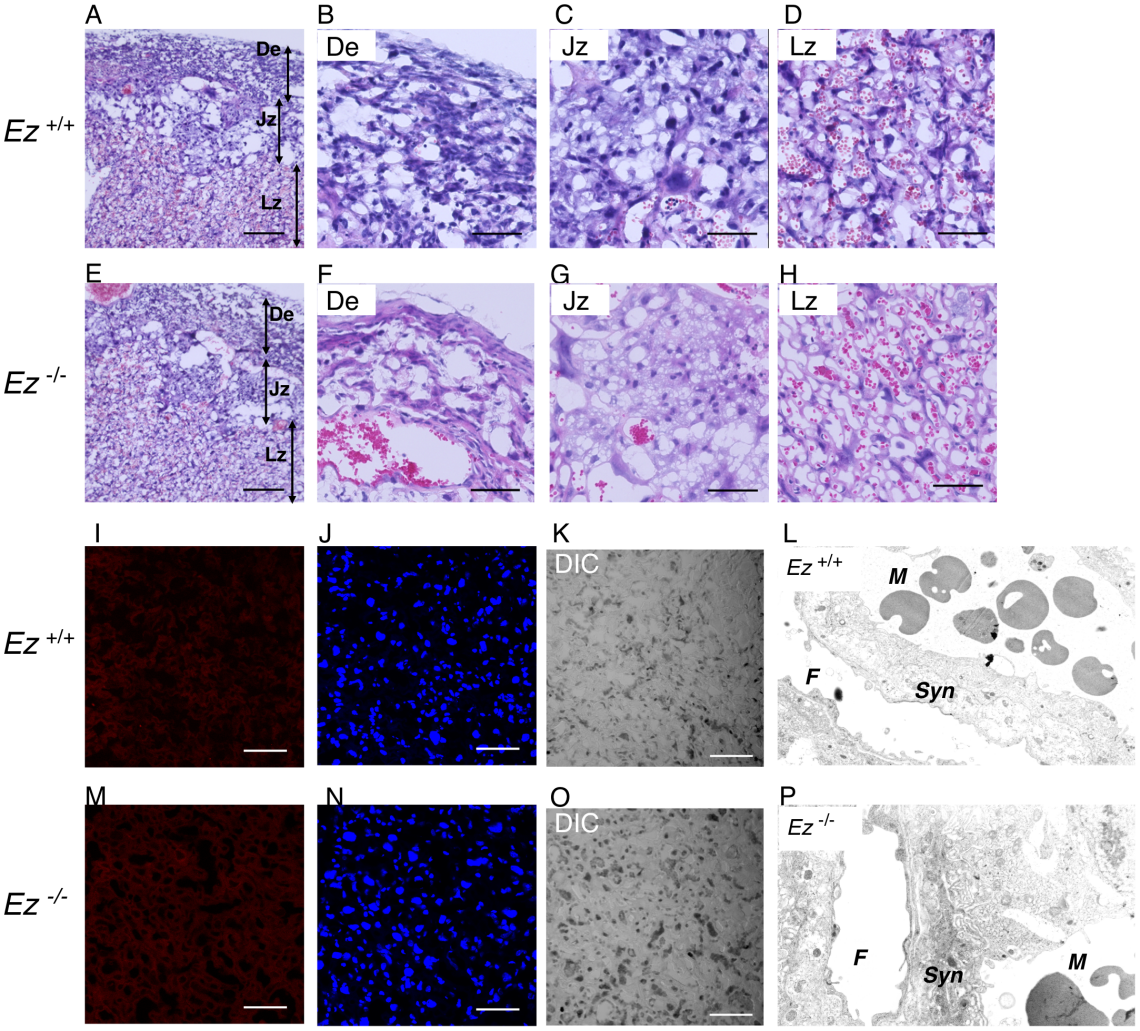
Morphological analysis of *Ez* ^−/−^ placenta and immunohistochemistry of ezrin. Placental sections prepared from *Ez* ^+/+^ (A, B, C, D) and *Ez* ^−/−^ (E, F, G, H) mice at gestational day 18.5 were morphologically analyzed by hematoxylin and eosin staining. Low magnification images (A, E) and higher magnification images for decidua (B, F), junctional (C, G) and labyrinth zone (D, H) are shown. Scale bars represent 200 µm (A, E) and 60 µm (B, C, D, F, G, H). De, decidua; Jz, junctional zone; Lz, labyrinth zone. TUNEL assay was conducted to visualize apoptotic cells in placental sections prepared from Ez^+/+^ (I, J, K) and Ez^−/−^ (M, N, O) mice at gestational day 18.5. DNA fragmentation caused by incubation with DNase I as a positive control was clearly detected (data not shown). TdT-mediated dUTP nick ends (red; I, M) and nuclei (blue; J, N) were visualized. Differential interference contrast (DIC) images are also shown (K, O). Scale bars represent 60 µm. Ultrastructure of *Ez* ^+/+^ (L) and *Ez* ^−/−^ (P) mice placenta stained with 1% uranyl acetate was viewed with an electron microscope. M, maternal blood side; F, fetal blood side; Syn, syncytiotrophoblast layer.

**Figure 3:**
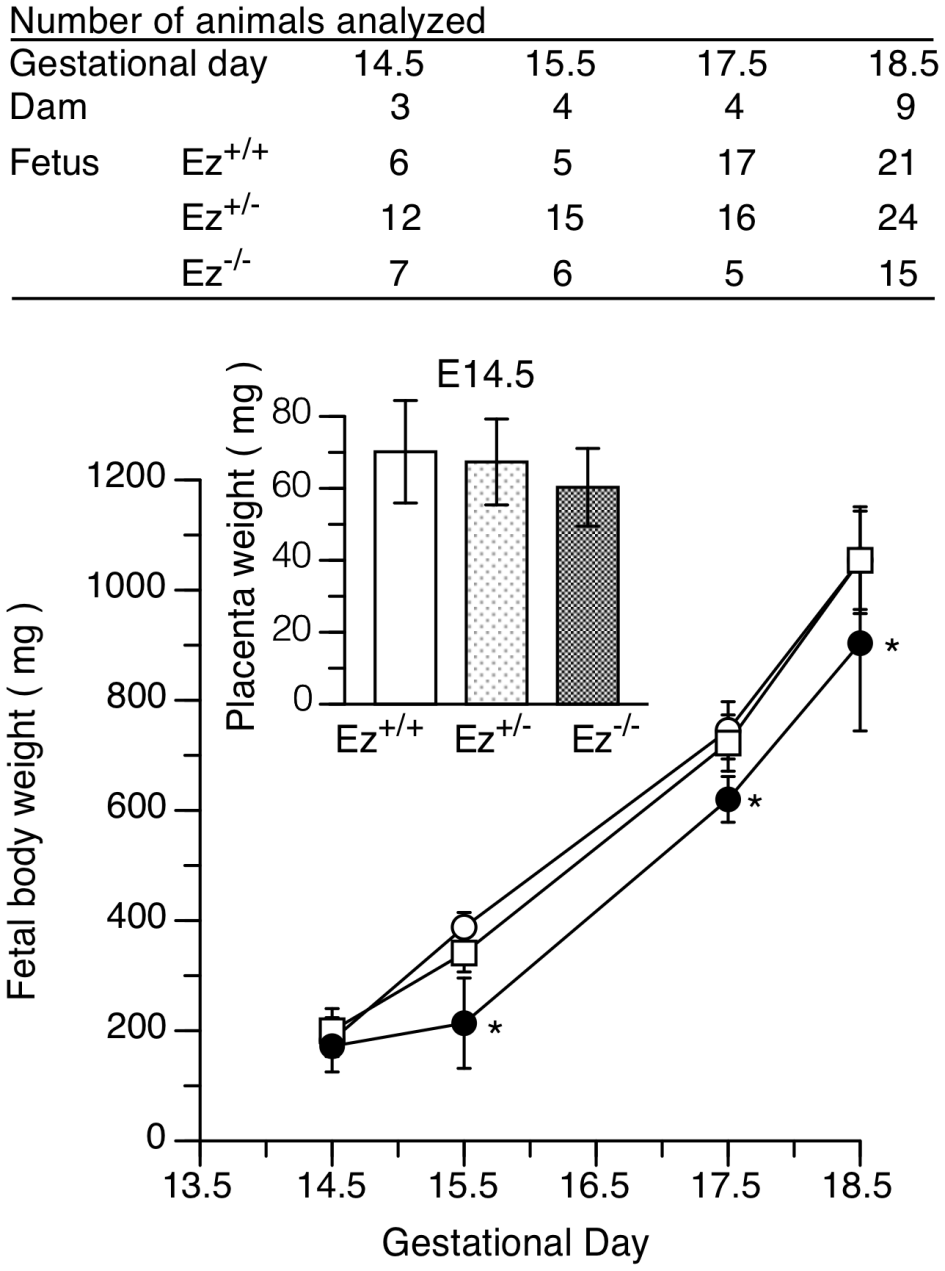
Fetal growth retardation of *Ez* ^−/−^ mice. Fetal body weights were measured from embryonic day 14.5 to 18.5 and data are shown by genotype: *Ez* ^+/+^ (○), *Ez* ^+/−^ (□) and *Ez* ^−/−^(•) mice. Placenta weights are shown in the inset. The number of fetuses of each genotype is shown in the upper panel. Data are presented as mean ± standard deviation. Statistical analysis was performed by one-way ANOVA with Tukey's multiple comparison test. *P* values less than 0.05 were regarded as significant and are indicated with an asterisk (*).

Knockout of the ezrin-encoding *Vil2* gene was confirmed by southern blotting (Figure 1A). We confirmed placental ezrin distribution using *Ez* ^+/+^ and *Ez* ^−/−^ mouse placenta. Immunofluorescence of ezrin was mainly stained in labyrinth zone of the *Ez* ^+/+^ mouse placenta (Figure 1B). The specificity of the staining was checked using *Ez* ^−/−^ mouse placenta. No clear signals were observed in *Ez* ^−/−^ mice placenta as expected (Figure 1C). Protein expression of ezrin was observed on the maternal membrane side of syncytiotrophoblast layers in the labyrinth zone of *Ez* ^+/+^ mouse placenta, while no immunofluorescence signals were observed at that site in *Ez* ^−/−^ mouse (Figure 1B, 1C). Placental sections prepared from *Ez* ^−/−^ at gestational day 18.5 were morphologically examined (Figure 2). We analyzed three major parts of placenta: decidua, junctional zone and labyrinth zone (Figure 2A, 2E). It has been uneasy to find morphological changes including distribution of giant cells (Figure 1B, 1C).in decidua (Figure 2B, 2F), junctional zone (Figure 2C, 2G) or labyrinth zone (Figure 2D, 2H) between *Ez* ^+/+^ and *Ez* ^−/−^ mice from these morphological analyses. We also investigated whether ezrin deficiency induced apoptosis, but few apoptotic cells were identified in the labyrinth zone of either *Ez* ^+/+^ or *Ez* ^−/−^ mouse placenta (Figure 2J, 2N), and differential interference contrast (DIC) microscopy revealed no marked differences (Figure 2K, 2O). Electron microscopic examination of syncytiotrophoblast layers indicated that the placenta of *Ez^−/−^* mice remained morphologically similar to that of wild-type mice (Figure 2L, 2P). Overall, these findings indicate that there are no marked structural differences in the placenta between *Ez* ^+/+^ and *Ez* ^−/−^ mouse. Nevertheless, fetal weights of *Ez^−/−^* mice from gestational day 15.5 to 18.5 were significantly lower than those of *Ez^+/+^* mice (Figure 3). Placenta weights of *Ez^−/−^* mice at both gestational day 14.5 and 18.5 were decreased by 10% on average compared to those of *Ez^+/+^* mice, but this difference was not statistically significant (Figure 3, inset).

### Metabolome analysis of *Ez^−/−^* mice fetal plasma and placenta

**Figure 4:**
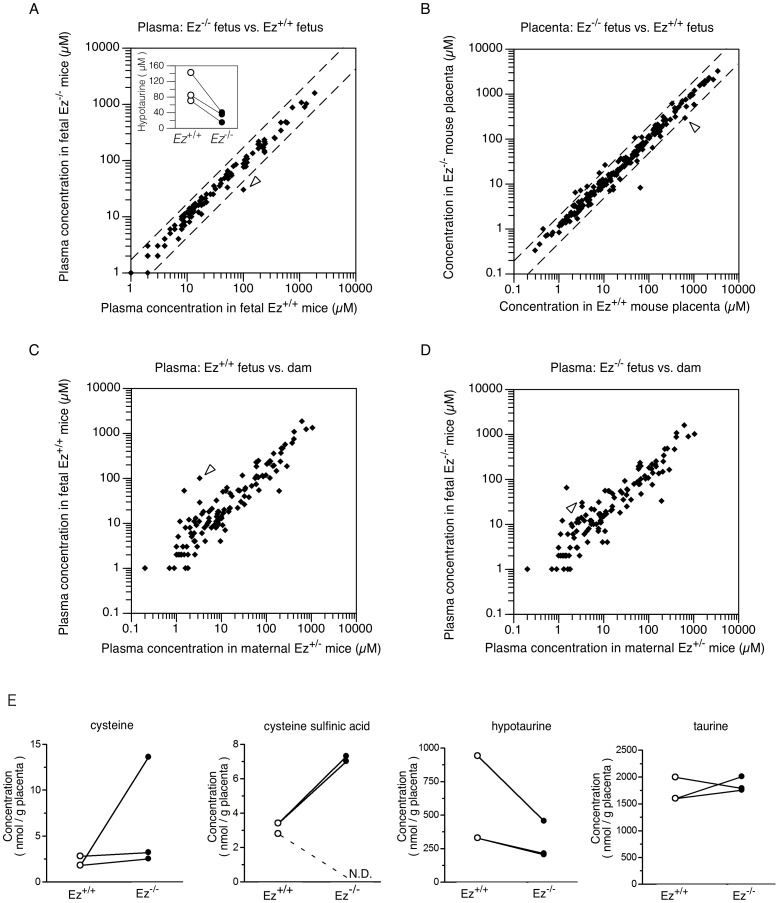
Effect of ezrin on physiological metabolites in fetal plasma and placenta. Plasma of three heterozygous (*Ez* ^+/−^) dams mated with *Ez* ^+/−^ mice, plasma from their fetuses (*Ez* ^+/+^ and *Ez* ^−/−^), and placenta were each isolated at gestational day 18.5, and subjected to CE-TOF/MS metabolomic analysis as described in the text. Scatter correlation graphs for fetal plasma (A) and placenta (B) of *Ez* ^+/+^ and *Ez* ^−/−^ mice, and for fetal and maternal plasma (C, D) are shown. Dotted lines indicate a 2-fold difference in concentration. Each dot represents the mean concentration of an individual metabolite. Open arrowheads indicate hypotaurine. Insets: plasma concentrations of hypotaurine in fetal *Ez* ^+/+^ and *Ez* ^−/−^ mice measured in metabolomic analysis were individually plotted. Lines link *Ez* ^+/+^ and *Ez* ^−/−^ littermates from the same *Ez* ^+/−^ mother.

To identify metabolites whose concentration is changed in *Ez^−/−^* mice, we carried out metabolome analysis of fetal plasma, placental extract, and maternal plasma by means of CE-TOF/MS. 123 and 190 metabolites were determined in mouse fetal plasma and placenta, respectively. The results are summarized in Figure 4. The only metabolite whose concentration was significantly lower in *Ez^−/−^* fetal plasma than in *Ez^+/+^* fetal plasma was hypotaurine, a precursor of taurine. The plasma levels of hypotaurine in *Ez^+/+^* and *Ez^−/−^* fetuses were 85.0±25.4 µM and 24.8±7.5 µM, respectively (*P*<0.05, Student's t-test) (Figure 4A). The paired t-test also showed a significant difference (*P*<0.05) of hypotaurine concentration between the genotypes (*Ez^+/+^* and *Ez^−/−^*) in the dams (Figure 4A, inset). The placental level of hypotaurine in *Ez* ^−/−^ fetus was also lower than that in *Ez* ^+/+^ fetus (Figure 4B). In *Ez^+/+^* fetal plasma, the concentration of hypotaurine was more than 20-fold higher than that in plasma of the dams (3.4±1.5 µM) (Figure 4C), while the difference in the case of *Ez* ^−/−^ fetuses and their dams was only 7-fold (Figure 4D). The biosynthetic precursors of hypotaurine are cysteamine and cysteine sulfinic acid, but these compounds were not detected in fetal plasma of either of the *Ez* genotypes. Hypotaurine itself is a precursor of taurine, but the taurine levels in *Ez^+/+^* and *Ez^−/−^* fetal plasma were similar. The placental levels of cysteamine, cysteine sulfinic acid and taurine showed no clear difference between *Ez^+/+^* and *Ez^−/−^*, though the level of hypotaurine in *Ez^−/−^* mouse placenta was slightly lower than that in *Ez^+/+^* (Figure 4E).

### Administration of hypotaurine to pregnant *Ez^+/−^* mice

**Figure 5:**
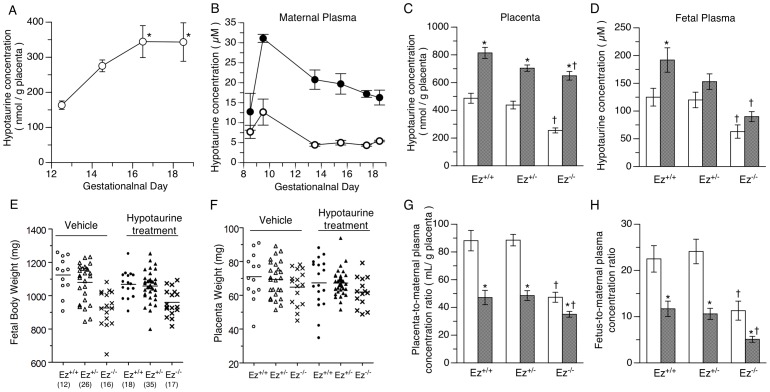
Effect of subcutaneous administration of hypotaurine to *Ez* ^+/−^ pregnant mice on the hypotaurine concentrations in fetus and placenta. Hypotaurine concentration in mouse placenta was measured during the course of late pregnancy (A). Data represents the mean and S.E.M of three determinations. Statistical analysis was conducted using one-way ANOVA with the Dunnett post-hoc test (control; day 12.5). Hypotaurine was continuously administered via an osmotic pump (12 mg/day/mouse) from gestational day 8.5 to pregnant *Ez^+/−^* female mice that had been mated with *Ez^+/−^* male mice. Plasma concentration of hypotaurine in maternal mice given hypotaurine (•) or vehicle (○) (B). Hypotaurine concentration in placenta (C) and fetus (D) of each genotype of *Ez* after administration of hypotaurine (hatched column) or vehicle (open column) to dams. Fetal body and placenta weights were measured at gestational day 18.5 (E, F). The number of fetuses of each genotype is shown in brackets (E). Statistical analysis was performed by one-way ANOVA with Tukey's multiple comparison test. *P* values less than 0.05 were regarded as significant and are indicated with an asterisk (*). The distribution of hypotaurine to placenta (G) and fetus (H) was evaluated by dividing the amount shown in panel C and D by the maternal plasma concentration at gestational day 18.5 shown in panel B. Comparisons between hypotaurine (hatched column) and vehicle (open column) administered groups or between genotypes were done by using Student's t-test. *P* values less than 0.05 were regarded as significant and are indicated with an asterisk (*) when compared to vehicle and a cross (†) when compared to *Ez^+/+^*.

Placental hypotaurine concentration in *Ez^+/+^ mice* increased from gestational day 12.5 to 16.5 and then remained at a constant level to term (Figure 5A). We next investigated the effect of hypotaurine supplementation in pregnant mice on the fetal and placental concentrations of hypotaurine and on fetal growth. Hypotaurine was continuously administered (12 mg/day/dam) via a subcutaneously implanted osmotic pump to pregnant *Ez* ^+/−^ mice that had been mated with *Ez* ^+/−^ male mice. The rate of hypotaurine supplementation was selected to increase the maternal hypotaurine level by 3-fold based on the results of a single dosing study (data not shown), and we expected that this would be sufficient to normalize the decreased level of hypotaurine in *Ez* ^−/−^ fetus. Plasma hypotaurine concentration in pregnant maternal mice treated with hypotaurine was indeed 3-fold higher than that pregnant maternal mice treated with vehicle (16.2 µM and 5.4 µM at gestational day 18.5, respectively) (Figure 5B). Hypotaurine levels in placental extract and fetal plasma of *Ez* ^−/−^ mice were almost half of those in *Ez* ^+/+^ mice, supporting the finding of lack of hypotaurine in *Ez* ^−/−^ mice that was implied by the results of metabolome analysis (Figure 5C, 5D). Hypotaurine concentration in placenta was significantly increased (1.6- to 2.5-fold) in each *Ez* genotype by continuous administration of hypotaurine (Figure 5C). Hypotaurine level in fetal plasma was increased 1.3- to 1.5-fold, though the increases in *Ez* ^+/−^ and *Ez* ^−/−^ fetuses were not statistically significant (Figure 5D). The plasma hypotaurine concentration in *Ez* ^−/−^ fetus administered with hypotaurine was still less than the level in vehicle-treated *Ez* ^+/+^ fetus (Figure 5D). Fetal body and placenta weights in hypotaurine-treated mice were similar to those in the vehicle-treated group (Figure 5E, 5F). To examine the placental and fetal supply of hypotaurine from maternal plasma, placenta-to-maternal plasma ratio and fetus-to-maternal plasma ratio were calculated. In the vehicle-treated group, the placenta-to-maternal plasma ratio of hypotaurine was more than 80 mL/g placenta in both *Ez* ^+/+^ and *Ez* ^+/−^ fetuses, while the ratio of *Ez*−/− fetus was significantly reduced at just over 40 mL/g placenta (Figure 5G). Similarly, the fetus-to-maternal plasma concentration ratio in *Ez* ^−/−^ mice was almost half of those in *Ez* ^+/+^ and *Ez* ^+/−^ (Figure 5H). Placenta-to-maternal plasma ratio and fetus-to-maternal plasma ratio in *Ez^+/+^* mice were decreased to 53% and 52%, respectively, by hypotaurine treatment (Figure 5G, 5H). *Ez* ^+/−^ mice showed similar characteristics to *Ez* ^+/+^ mice (Figure 5G, 5H). In contrast, the placenta-to-maternal plasma and fetus-to-maternal plasma ratio in *Ez* ^−/−^ mice were decreased to 74% and 45%, respectively, by hypotaurine treatment (Figure 5G, 5H).

## Discussion

The present study indicates that ezrin is responsible for fetal growth in late gestation. Hypotaurine was the only metabolite significantly changed in *Ez*−/− fetal mice and ezrin plays a role in hypotaurine transport systems in the placenta, which contribute to fetal hypotaurine level. Fetal hypotaurine is at least partly is supplied from mother via placental hypotaurine transport system(s).

Ezrin was primarily expressed in the labyrinth zone of placenta (Figure 1B), and was predominantly localized at the apical membrane of syncytiotrophoblast in placenta of *Ez* ^+/+^ fetal mice (Figure 1B), in accordance with previous observations in rat placenta [14]. Intrauterine growth retardation was observed at gestational day 15.5 and later in *Ez^−/−^* mice (Figure 3). Morphological analyses revealed little structural impairment in the labyrinth zone of *Ez^−/−^* placenta, or in the junctional zone and decidua (Figure 2). Apoptotic cells were rare in *Ez^−/−^* placenta (Figure 2). In addition, the placental weight of *Ez^−/−^* mice was not significantly lower than that of *Ez* ^+/+^ mice (Figure 3). These results collectively suggest that ezrin expressed in placenta does not have a major role in placental structure and development. Thus, we hypothesized that knockdown of ezrin results in functional impairment of nutrient transporter(s) in the apical membrane of syncytiotrophoblast in late pregnancy, thereby reducing fetal growth without causing morphological change.

Metabolome analysis showed that hypotaurine concentration was decreased in fetal plasma and placenta of *Ez^−/−^* littermates in comparison with *Ez^+/+^* littermates (Figure 4), and excluded the possibility that metabolic changes in *Ez^+/−^* dams contributes to the lack of hypotaurine in *Ez^−/−^* fetus. Hypotaurine, a precursor of taurine, is synthesized from cysteine through cysteine sulfinic acid or cysteamine. Hypotaurine-synthesizing and -metabolizing enzymes, such as cysteine sulfinic acid decarboxylase, cysteamine dioxygenase and hypotaurine oxidase, are expressed in the cytosolic or mitochondrial compartment [19]–[21]. Since ezrin is a scaffold protein of plasma membrane transporters and is localized at the apical membrane of syncytiotrophoblast (Figure 2), it is unlikely that ezrin deficiency would affect hypotaurine-synthesizing enzymes. Instead, decreased level of hypotaurine in *Ez^−/−^* fetus might be caused indirectly by deficiency of hypotaurine precursors or directly by deficiency of hypotaurine transport from mother to fetus. We found no significant changes in biosynthetic precursors (Figure 4), suggesting that hypotaurine is directly supplied from mother to fetus across the placenta. These results are consistent with the idea that ezrin plays a role in hypotaurine transport function in syncytiotrophoblasts.

Hypotaurine concentration in placenta increased from mid to term gestation (Figure 5A), implying that hypotaurine transport activity was induced during the later gestational period. These observations led us to hypothesize that hypotaurine supplementation of dams might satisfy the hypotaurine demand of *Ez* ^−/−^ fetal mice in late gestation. We selected a rate of hypotaurine administration that we expected to achieve a 3-fold increase in maternal plasma hypotaurine, as shown in Figure 5B, anticipating a parallel (3-fold) increase in fetus hypotaurine. First, lack of hypotaurine in *Ez* ^−/−^ mice was confirmed by LC/MS/MS (Figure 5C, 5D). The plasma hypotaurine level in *Ez* ^+/+^ fetal plasma was increased by hypotaurine administration to the mother, but by only 1.5-fold, which was less than expected (Figure 5D). This result at least indicates that hypotaurine in the fetus is partly supplied from maternal plasma. In contrast, the increase of hypotaurine level in *Ez^−/−^* fetal mice was not significant and the level remained lower than that in *Ez^+/+^* fetal mice treated with vehicle (Figure 5D). This result indicates that ezrin plays a critical role in transport of hypotaurine to the fetus. Placental concentration of hypotaurine was apparently increased by hypotaurine treatment in all genotypes (Figure 5C). Contamination of placenta with maternal blood could not account for this, because hypotaurine was highly concentrated in placenta compared to maternal plasma (Figure 5C, 5G). To address the transport process from mother to placenta, we analyzed placenta-to-maternal plasma concentration ratio of hypotaurine. The placenta-to-maternal plasma concentration ratio in *Ez^−/−^* mice was almost half of that in *Ez* ^+/+^ mice in the vehicle treatment group (Figure 5G). In *Ez* ^+/+^ mice, the ratio was 50% decreased by maternal hypotaurine treatment, becoming similar to that of *Ez^−/−^* mice (Figure 5G). On the other hand, in *Ez^−/−^* mice the ratio was only slightly decreased by maternal hypotaurine treatment (Figure 5G). These results suggest that hypotaurine is taken up into placenta from maternal plasma via a saturable process and that disruption of ezrin in placenta decreases the saturable uptake activity. Although the transporter responsible for hypotaurine uptake from maternal plasma to placenta has not been identified yet, the results obtained in this study may be explained by the hypothesis that a hypotaurine transporter with an affinity for hypotaurine similar to the plasma concentration (5–20 µM) is expressed on the apical membrane of syncytiotrophoblast, and is functionally regulated by ezrin.

However, fetal growth retardation of *Ez^−/−^* mice was not ameliorated by hypotaurine treatment (Figure 5E). There are two possible explanations for this: 1) fetal growth retardation in *Ez^−/−^* mice was independent of hypotaurine deficiency, 2) hypotaurine administration was insufficient to reverse the lack in the fetus. As to the first possibility, metabolomic screening showed that only hypotaurine was significantly changed in the *Ez^−/−^* fetus (Figure 4A). Therefore this possibility seems unlikely on the assumption that metabolic changes are involved in the fetal growth retardation of *Ez* ^−/−^ mice. The second possibility cannot be excluded. However, administration of hypotaurine at a higher concentration would not be effective, since uptake of hypotaurine from mother to placenta was saturated under the conditions of the present study (Figure 5E). It would be interesting to examine the effects of other compounds that might physiologically compensate for the decrease of hypotaurine.

Hypotaurine is a biosynthetic precursor of taurine. A relationship between taurine deficiency and IUGR has been reported, and thus impaired placental function of taurine transporter is at least one of the potential causes of IUGR [22]. Our current data imply that hypotaurine transporter plays a role in supplying hypotaurine to placenta and fetus, but the hypotaurine level in fetus seems unlikely to be linked to taurine level (Figure 4). Hypotaurine itself is an antioxidant [23] and reacts with hydroxyl radical and singlet oxygen [23]–[25]. In liver, it shows a protective effect against ischemic injury [26]. Thus, other scavengers of reactive oxygen species (ROS) might compensate at least in part for lack of hypotaurine.

In the present study, we have shown the fetal growth retardation and lack of hypotaurine in *Ez^−/−^* mice. Expression of ezrin in microvillous membrane of human IUGR placenta was similar to that of normal placenta, indicating that the frequency of ezrin deficiency seems to be rare in the placenta of pregnancy complicated with IUGR [27]. The function of ezrin is regulated by phosphorylation and it is still unclear that phosphorylated ezrin level in the placenta with IUGR. It is also possible that ezrin-associated protein causes malfunction of placenta such as fetal hypotaurine deficiency. There are no reports showing fetal hypotaurine level in human. The relationship of fetal hypotaurine level and placental expression both of ezrin and a potential hypotaurine transporter should be clarified in the future studies.

In summary, fetal growth retardation was observed in *Ez* ^−/−^ mice, but no marked morphological damage was observed. Metabolome analysis revealed that hypotaurine is the only component, among those determined, that exhibits a lower concentration in fetal plasma and placenta of *Ez^−/−^* mice, as compared with *Ez^+/+^* mice. The increase of hypotaurine in maternal plasma was not parallel to that in fetal plasma, and the results indicate that *Ez^−/−^* mice lack a saturable hypotaurine uptake process from mother to fetus. These results suggest that ezrin is involved in regulation of a putative transporter that mediates mother-to-placenta transfer of hypotaurine.
